# Fra-1 is a key driver of colon cancer metastasis and a Fra-1 classifier predicts disease-free survival

**DOI:** 10.18632/oncotarget.6454

**Published:** 2015-12-03

**Authors:** Sedef Iskit, Andreas Schlicker, Lodewyk Wessels, Daniel S. Peeper

**Affiliations:** ^1^ Department of Molecular Oncology, The Netherlands Cancer Institute, Plesmanlaan, Amsterdam, The Netherlands; ^2^ Department of Molecular Carcinogenesis, The Netherlands Cancer Institute, Plesmanlaan, Amsterdam, The Netherlands

**Keywords:** colon cancer, metastasis, Fra-1, in vivo, Wnt

## Abstract

Fra-1 (Fos-related antigen-1) is a member of the AP-1 (activator protein-1) family of transcription factors. We previously showed that Fra-1 is necessary for breast cancer cells to metastasize *in vivo*, and that a classifier comprising genes that are expressed in a Fra-1-dependent fashion can predict breast cancer outcome. Here, we show that Fra-1 plays an important role also in colon cancer progression. Whereas Fra-1 depletion does not affect 2D proliferation of human colon cancer cells, it impairs growth in soft agar and in suspension. Consistently, subcutaneous tumors formed by Fra-1-depleted colon cancer cells are three times smaller than those produced by control cells. Most remarkably, when injected intravenously, Fra-1 depletion causes a 200-fold reduction in tumor burden. Moreover, a Fra-1 classifier generated by comparing RNA profiles of parental and Fra-1-depleted colon cancer cells can predict the prognosis of colon cancer patients. Functional pathway analysis revealed Wnt as one of the central pathways in the classifier, suggesting a possible mechanism of Fra-1 function in colon cancer metastasis. Our results demonstrate that Fra-1 is an important determinant of the metastatic potential of human colon cancer cells, and that the Fra-1 classifier can be used as a prognostic predictor in colon cancer patients.

## INTRODUCTION

Metastasis is the main reason for many solid tumors to be life-threatening. The metastatic cascade involves several steps, ranging from dissemination from the primary tumor to growth at a secondary site. The acquisition of metastatic capability by tumor cells can be associated with Epithelial-Mesenchymal Transition (EMT). Upon EMT, tumor cells are able to invade through the basement membrane of the primary tissue and stroma, and to enter the blood circulation. They often become anoikis resistant, which allows them to survive in the absence of attachment. Finally, they associate with the endothelium and extravasate to a secondary tissue. For outgrowth at secondary sites, the newly formed tumor foci need to induce angiogenesis [1, 2]. Metastases are often difficult to cure because they can be widespread, affecting tissue function, and they are usually resistant to conventional therapies. Furthermore, intervention of metastatic cancer progression is rarely efficient due to lack of early detection methods. Therefore, it is crucial to predict metastatic potential of disease and to target metastasis.

One of the well-known regulators of metastasis is the Activator Protein 1 (AP-1) complex. AP-1 is a family of transcription factors regulating a broad spectrum of cellular processes including proliferation, migration and invasion [3]. AP-1 dimers are formed by Fos (c-FOS, FOSB, Fra-1, Fra-2), Jun (c-JUN, JUNB, JUND), ATF and MAF protein families. AP-1 members are encoded by immediate early genes that are rapidly activated and deactivated in response to a wide range of stimuli. Although some AP-1 components have been reported to act as tumor suppressors, AP-1 complexes are mostly known for their ability to induce oncogenic transformation among other processes such as proliferation, apoptosis, invasion and angiogenesis [4]. c-Fos, c-Jun and Fra-1 are among the AP-1 components whose overexpression correlate with poor prognosis in several types of malignancies including ovarian, lung, and breast cancers [5-7].

AP-1 is regulated by the Ras/Raf/MEK/ERK [8], [9] and the Wnt [10] pathways. The Wnt pathway is often deregulated in colon cancer as a result of activating mutations in beta-catenin (CTNNB1) or inactivating mutations in adenomatous polyposis coli (APC), which is a negative regulator of beta-catenin. Wnt signaling is not only critical for developmental and oncogenic characteristics like proliferation, survival, and differentiation but also drives metastasis-related processes such as migration and cell polarity [11]. Previous reports have shown that the Wnt pathway negatively regulates Fos and FosB expression, whereas it increases Fra-1 mRNA levels in mouse epithelial cells [12]. Moreover, non-canonical Wnt signaling activates AP-1 through TCF binding to c-Jun in human colon cancer cells [13].

Fra-1 is one of the AP-1 transcription factors; it lacks a transactivation domain and has therefore a weak transforming activity. It forms heterodimers with Jun family members in order to activate target gene transcription. We and others have shown that Fra-1 promotes metastasis through various molecules: ADORA2B [7] in breast cancer, MMPs in breast cancer [14] and in lung epithelial cells [15], CD44 in mesothelioma [16], AXL in bladder cancer [17], FAK and EZH2 in colon cancer [18], [19].

Colorectal cancer (CRC) is among the most common cancers and one of the leading causes of cancer-related deaths worldwide. Traditional classification divides CRC into four main stages based on the local extent of the tumor, with three subtypes of stage III tumors based on the number of cancer-positive nodes [20]. However, CRC is more heterogeneous than the categories used in the clinic with regard to progression, recurrence, metastasis and therapy response[21]. In the present study, we investigated the role of Fra-1 in colon cancer progression *in vivo* and the clinical impact of Fra-1 on disease outcome.

## RESULTS

### Fra-1 is not critically required for proliferation of colon cancer cells *in vitro*

**Figure 1:**
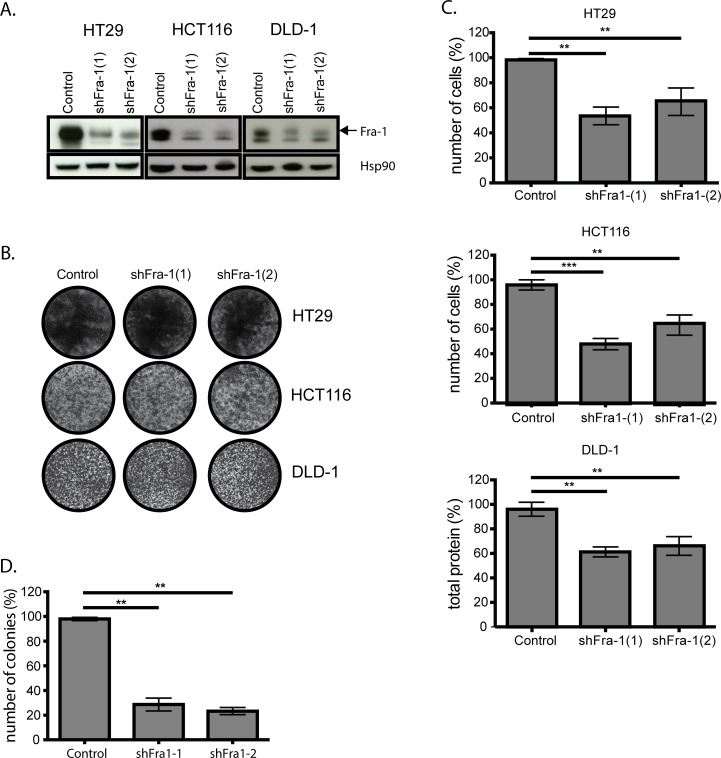
Fra-1 is not critically required for proliferation of colon cancer cells *in vitro* **A**. Fra-1 depletion upon lentiviral transduction of two independent shRNAs. **B**. HT29 and HCT116 cells with or without Fra-1 knockdown were seeded into 6-well plates (30000 cells/well). The plates were stained with crystal violet after 7 days. **C**. HT29 cells with or without Fra-1 knockdown were seeded in duplicates in 0,3% agar suspension on top of a 1% agar base in 6-well plates at 24000 cells/well. After three weeks, colonies were stained with crystal violet and counted by Image J software (n=3). **D**. 0.4*10^6^ cells were seeded into 6-well ultra-low-attachment plates in duplicate. The cells were harvested at day 6, trypsinized, resuspended and counted. Results presented are the combination of three experiments. Error bars represent SEM. Statistics: One-Way ANOVA. * *p* < 0.05, ** *p* < 0.01, *** *p* < 0.001.

As we have previously shown that Fra-1 is largely dispensable for human breast cancer cell growth *in vitro* but crucial for their ability to metastasize *in vivo* [7], we decided to investigate whether Fra-1 has a similar role in human colon cancer. Fra-1 was stably depleted in HT29, HCT116 and DLD-1 cells by two independent shRNAs (Figure 1A). There was no difference in proliferation rates of Fra-1-depleted cells and control cells on 2D culture plates (Figure 1B). However, we found a 30-50% decrease in the number of cells surviving under anoikis-inducing conditions (Figure 1C) and a three-fold decrease in the number of colonies formed by Fra-1 deficient HT29 cells in soft agar (Figure 1D). Colo205 cells, which have low endogenous Fra-1 expression levels, successfully formed colonies in soft agar and survived in anoikis-inducing conditions; Fra-1 overexpression caused a mild but significant increase in the number cells in both cases (Suppl. Fig. 1A-1C). Thus, Fra-1 is neither critically required for 2D nor 3D proliferation *in vitro*.

### Fra-1 is largely dispensable for primary colon tumor growth *in vivo*

**Figure 2:**
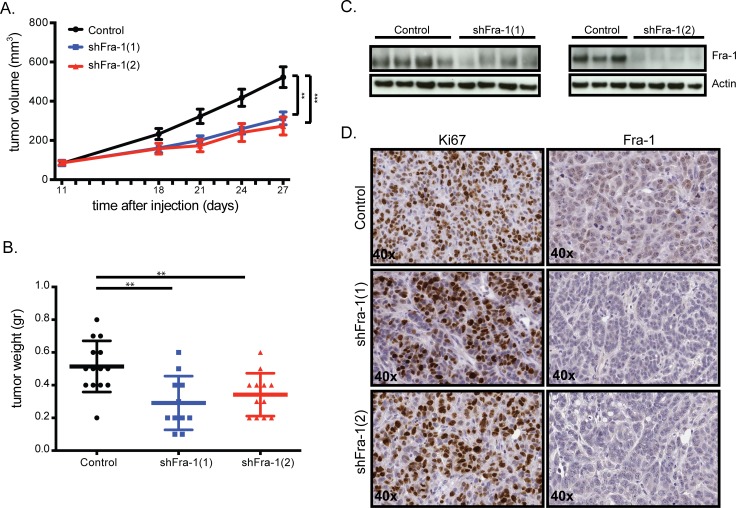
Fra-1 is largely dispensable for primary colon tumor growth *in vivo* **A**. 0,5*10^6^ HT29 cells with or without Fra-1 knockdown were mixed 1:1 with matrigel and injected subcutaneously into severely immune compromised (NOD/SCID IL2γ) mice. Tumor growth was measured by a caliper at indicated time points (n_control_=13, n_shFra1-1_=12, n_shFra1-2_=13). **B**. The weight of tumors harvested at the end time point of two independent experiments combined. **C**. Western blot showing Fra-1 levels in tumors harvested from mice injected with control or Fra-1-depleted HT29 cells. **D**. Tumors harvested at the end of the experiment were analyzed for the expression of Ki67 and Fra-1 by immunostaining (40X). Error bars represent SEM. Statistics: One-Way ANOVA. * *p* < 0.05, ** *p* < 0.01, *** *p* < 0.001.

In order to assess the role of Fra-1 in *in vivo* tumor growth, we next injected control and Fra-1-depleted HT29 cells subcutaneously into severely immune-compromised (NOD/SCID IL2gamma, NSG) mice. Fra-1-depleted tumors grew approximately two-fold slower than control tumors (Figure 2A-2B). Immunohistochemistry staining and western blots showed that Fra-1 levels were still low in these tumors at the end of the experiment (Figure 2C-D), indicating that there is no selective pressure to lose Fra-1 shRNAs during tumor progression. These data show that although Fra-1 contributes somewhat to the expansion of colon cancer tumors *in vivo*, it is not strictly required.

### Fra-1 is crucial for efficient metastatic spread of colon cancer cells

**Figure 3:**
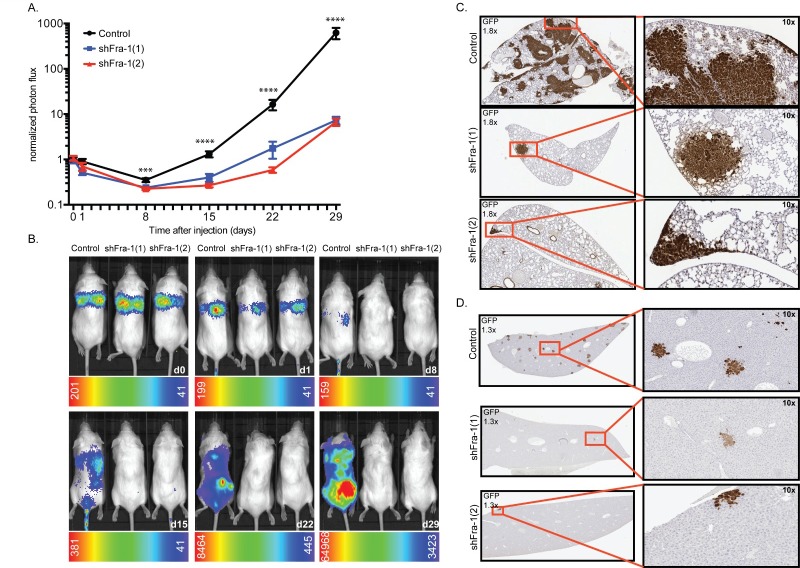
Fra-1 is crucial for efficient metastatic spread of colon cancer cells **A**. 0,5*10^6^ HT29 cells were injected intravenously into NSG mice. Tumor expansion was followed by IVIS from day0 onwards. Photon flux from two independent experiments was combined. n = 6 mice/group/experiment. Error bars represent SEM. Statistics: One-Way ANOVA * *p* < 0.05, **p < 0.005,****p < 0.0001 **B**. Representative images of mice at each time point. **C.**-**D**. GFP staining showing tumor foci in lungs and liver.

We and others have implicated Fra-1 as an important determinant of the metastatic capacity of cancer cells, which is associated with its ability to induce EMT and with clinical outcome [7, 22]. In order to determine the role of Fra-1 in colon cancer metastasis *in vivo*, we injected Fra-1-depleted HT29 cells intravenously into NSG mice and monitored tumor expansion in time via a luciferase-dependent non-invasive *in vivo* imaging system. Whereas mice injected with cells carrying a control construct showed a substantial number of tumor foci distributed all over the body, tumor burden was sharply reduced in mice injected with Fra-1-depleted cells (Figure 3A-3B). 29 days after injection, control mice had a saturated luciferase signal accompanied by severe weight loss (Suppl. Fig. 2A). At this time point, the average difference between control mice and mice injected with Fra-1-depleted cells was 206-fold.

At autopsy, multiple macroscopic tumors were observed on the subcutaneous skin and peritoneal wall as well as several organs of the control mice such as lung, spine, kidneys, ovaries, lymph nodes, skin and muscles in the extremities (Suppl. Fig. 2B). Immunohistochemical staining further showed foci in the liver, bones and brain. Much fewer tumors were observed in the mice injected with Fra-1-depleted cells, both macroscopically and by immunostaining (Figure 3C-3D). Importantly, and in contrast to our observations for primary tumor growth, in a great majority of the cases, the tumor foci formed by Fra-1-depleted cells were positive for Fra-1, sometimes in a heterogeneous fashion (Suppl. Fig. 2C, 2D, 2E). We observed a similar pattern in HCT116 cells, which metastasize preferentially to the liver: Fra-1- depleted HCT116 cells formed significantly fewer and smaller foci in the liver upon intravenous injection (Suppl. Fig. 1D). Together, these results demonstrate that Fra-1 is critical for the metastatic spread of colon cancer cells *in vivo*, yet expendable for primary tumor growth.

### Acute Fra-1 depletion impairs growth of established metastatic foci

**Figure 4:**
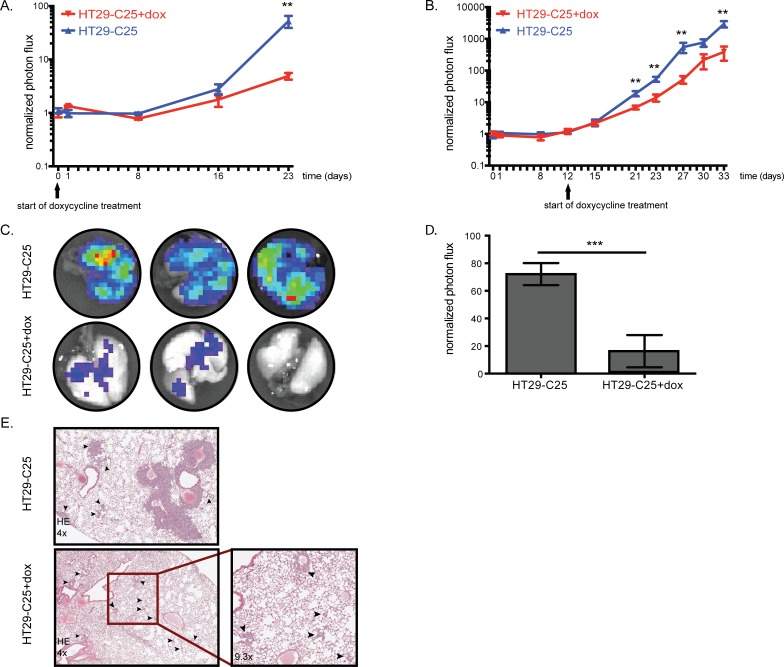
Acute Fra-1 depletion impairs growth of established metastatic foci **A**.-**B**. 0,5*10^6^ HT29-C25 cells were intravenously injected into NSG mice. Growth curves of intravenously injected HT29-C25 cells with or without doxycycline treatment starting at day0 or day12. **C**.-**D**. The mice in the experiment shown in B were injected with 15mg/kg luciferin five minutes before sacrifice. The lungs were harvested and imaged in luciferin-containing PBS and quantified. E. Hematoxylin and eosin staining of representative lung sections from mice of experiment shown in B, treated with doxycycline or not. The arrowheads point to the tumor foci. Error bars represent SEM. Statistics: Non-parametric student's *t*-test. **p < 0.005,***p < 0.001.

The results obtained with cells lacking Fra-1 expression suggest an important contribution of Fra-1 to the metastatic potential of colon cancer cells. From a clinical point of view, it would be more relevant to determine the impact of Fra-1 depletion on tumors that have already been established, rather than to prevent outgrowth. Therefore, we decided to investigate whether acute loss of Fra-1 affects the growth of established tumor foci. This system also allowed us to exclude the potential bias where one group of cells may not survive the injection procedure or the mechanical stress caused by the blood circulation. We used an inducible tet-on system enabling us to deplete Fra-1 on doxycycline administration via the drinking water of the mice (Suppl. Fig 3A). To ensure a homogeneous level of downregulation of Fra-1 upon doxycycline treatment, we generated a cell clone (HT29-C25) harboring the tet-on construct. The mice were injected intravenously with control or HT29-C25 cells and each group was randomized into two sub-groups at the day of injection. One group continuously received doxycycline in drinking water from day 0 onwards after the inoculation of tumor cells, whereas the other was mock-treated. The total tumor burden was reduced 56-times in HT29-C25-injected mice upon doxycycline treatment (Figure 4A), whereas mice injected with control cells had no difference in luciferase signal until the end of the experiment (Suppl. Fig. 3B). This result indicates that when Fra-1 knockdown is induced after the initial seeding of tumor cells upon intravenous inoculation, Fra-1 is required for tumor outgrowth.

Next, Fra-1 depletion was induced when the tumor burden started increasing (after an initial drop as judged by luciferase imaging). Twenty days after treatment, Fra-1 depletion caused an eight-fold reduction in tumor burden (Figure 4B). Once again, the rate of tumor development was the same in the mice injected with control cells regardless of doxycycline treatment (Suppl. Fig. 3C). Similar to the previous experiment (Figure 3), mice injected with control cells developed tumors in a broad range of organs, but there were very few macroscopically detectable tumors in the HT29-C25-injected mice on doxycycline treatment. Also similar to the previous experiment, tumors harvested at the end of the experiment showed varying levels of Fra-1 suggesting that some tumors were formed by Fra-1-proficient cells (Suppl. Fig. 3D). As assessed by the luciferase signal, doxycycline-treated mice have approximately five times less burden in their lungs compared to mock-treated mice (Figure 4C-4D). These mice had not only fewer but also smaller tumor foci in their lungs (Figure 4E). Altogether, these data suggest that Fra-1 is essential also for growth and expansion of established (micro)metastases of colon cancer cells.

### Fra-1-regulated gene signature is a prognostic classifier in colon cancer

**Figure 5:**
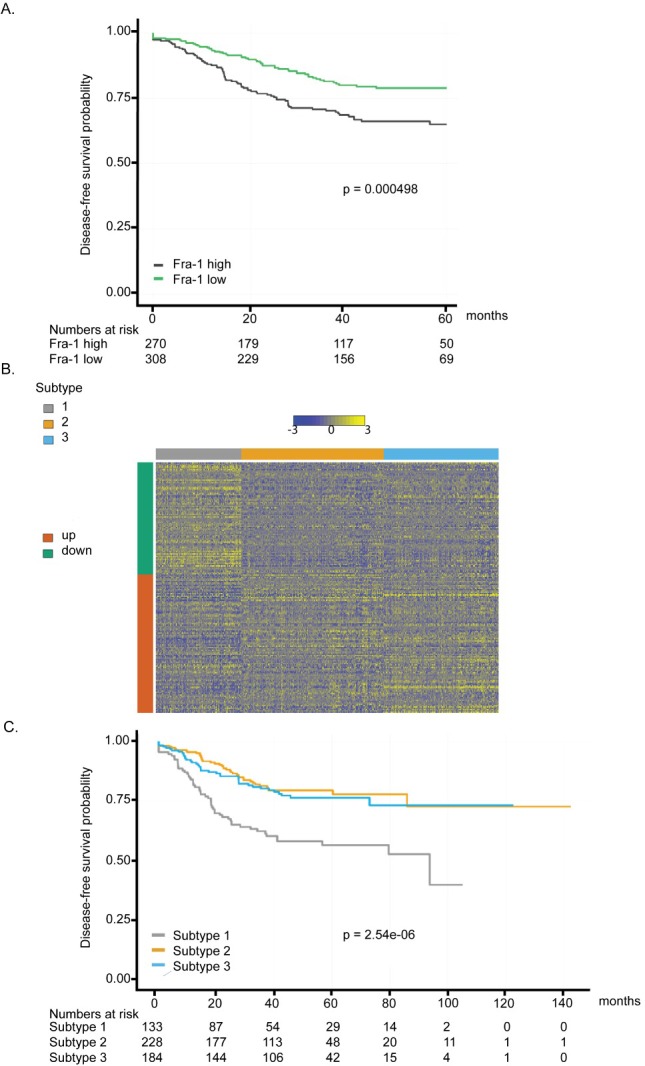
Fra-1-regulated gene signature is a prognostic classifier in colon cancer **A**. Disease free survival (DFS) analysis based on *FOSL1* expression. Samples were split according to lower or higher than average expression of *FOSL1*. Patients with low expression of *FOSL1* exhibited significantly longer DFS than patients with higher expression. **B**. Heat map of the gene expression of the Fra-1 signature. Gene expression is shown as color gradient from blue (low expression) to yellow (high expression). The color bar on the left side indicates direction of regulation in Fra-1 knock-down cells. Color bars on top of the heat map show sample stage, source data set and Fra-1KD signature cluster, in this order. **C**. Disease-free survival curve for the three subtypes resulting from hierarchical clustering with the Fra-1 signature. Subtype 1 has significantly shorter DFS than subtypes 2 and 3, which show no survival difference.

Based on these findings, which are consistent with, and extend, those of others [18], [23], [24], Fra-1 acts as an important pro-metastatic factor in colon cancer. Since metastatic relapse is a major reason of cancer-related deaths, we asked whether we could stratify colon cancer patients based on Fra-1 expression levels, similar to what we have shown recently for breast cancer [7]. The prognostic value of Fra-1 was assessed by correlating *FOSL1* mRNA levels (encoding Fra-1) in colon cancer patient samples to disease-free survival in five gene expression datasets. We observed that patients with tumors showing *FOSL1* expression higher than median levels had a significantly worse prognosis in the first five years after treatment or surgery (Figure 5A).

However, Fra-1 is not an ideal drug target due to the absence of a catalytic site that can be readily targeted by a small molecule. The lack of an available inhibitor against Fra-1 prompted us to search for critical downstream targets of Fra-1 that are involved in metastasis. We compared the expression profiles of control and Fra-1-depleted HT29 cells by RNA sequencing and selected the genes that are significantly regulated by Fra-1. This classifier contains a total of 199 genes, 88 of which are positively regulated by Fra-1 and 111 negatively (Suppl. Table 1).

According to non-negative matrix factorization (NMF) analysis, colon cancer patients could be divided into three prognostic groups based on the expression levels of Fra-1-regulated genes. The heat map demonstrates that the genes that were positively regulated by Fra-1 are overexpressed in patients in subtype 1 and not in subtypes 2 and 3. On the other hand, genes that were negatively regulated by Fra-1 have lower expression levels in patients in cluster 1 (Figure 5B), independently of tumor stage or dataset (Suppl. Fig. 4). A Kaplan-Meier analysis showed that subtypes 2 and 3 are good prognosis groups. They only slightly differ from each other in the initial survival rates but in the long term have a similarly good prognosis. subtype 1, on the other hand, has a significantly worse disease-free survival compared to the other two groups as well as poorer disease-specific and overall survival (Figure 5C, Suppl. Fig. 5). In a Cox proportional hazards model stratified for gender and stage, subtypes 2 and 3 showed significantly better disease-free survival (HR = 0.43, *p* = 4.42*10^−5^ and HR = 0.51, *p* = 0.001) than subtype 1. Analyzing each stage separately, we found similar effects for each stage albeit with different effect size (Table 1). A comparable pattern was observed with disease-specific and overall survival analysis with the exception of stage 3 patients in subtype 3 in case of overall survival (Suppl. Table 2a, 2b). These data suggest that overexpression of genes positively regulated by Fra-1 is correlated with poor outcome, whereas the expression of genes negatively regulated by Fra-1 is associated with better outcome. Therefore, our Fra-1 classifier has prognostic power to predict the clinical outcome of colon cancer patients.

**Table 1:** Cox proportional hazards model estimating hazard ratios for disease-free survival for the subtypes stratified for gender and tumor stage

		N(n)	HR	95% CI	p-value
**Subtype**	**1**	78 (55)	1		
	**2**	228 (42)	0.43	0.28-0.64	4.42*10^−5^
	**3**	184 (40)	0.51	0.33-0.77	0.001

Stage 2					
		N(n)	HR	95% CI	p-value
**Subtype**	**1**	66 (19)	1		
	**2**	126 (12)	0.2999	0.15 - 0.68	0.0011
	**3**	97 (15)	0.4902	0.25 - 0.97	0.0393

Stage 3					
		N(n)	HR	95% CI	p-value
**Subtype**	**1**	52 (27)	1		
	**2**	65 (21)	0.5213	0.29 - 0.92	0.0253
	**3**	60 (21)	0.6612	0.37 - 1.17	0.1569

Stage 4					
		N(n)	HR	95% CI	p-value
**Subtype**	**1**	8 (8)	1		
	**2**	11 (8)	0.37	0.13 - 1.03	0.0572
	**3**	8 (4)	0.19	0.05 - 0.71	0.0132

### Fra-1 regulates the Wnt pathway

**Figure 6:**
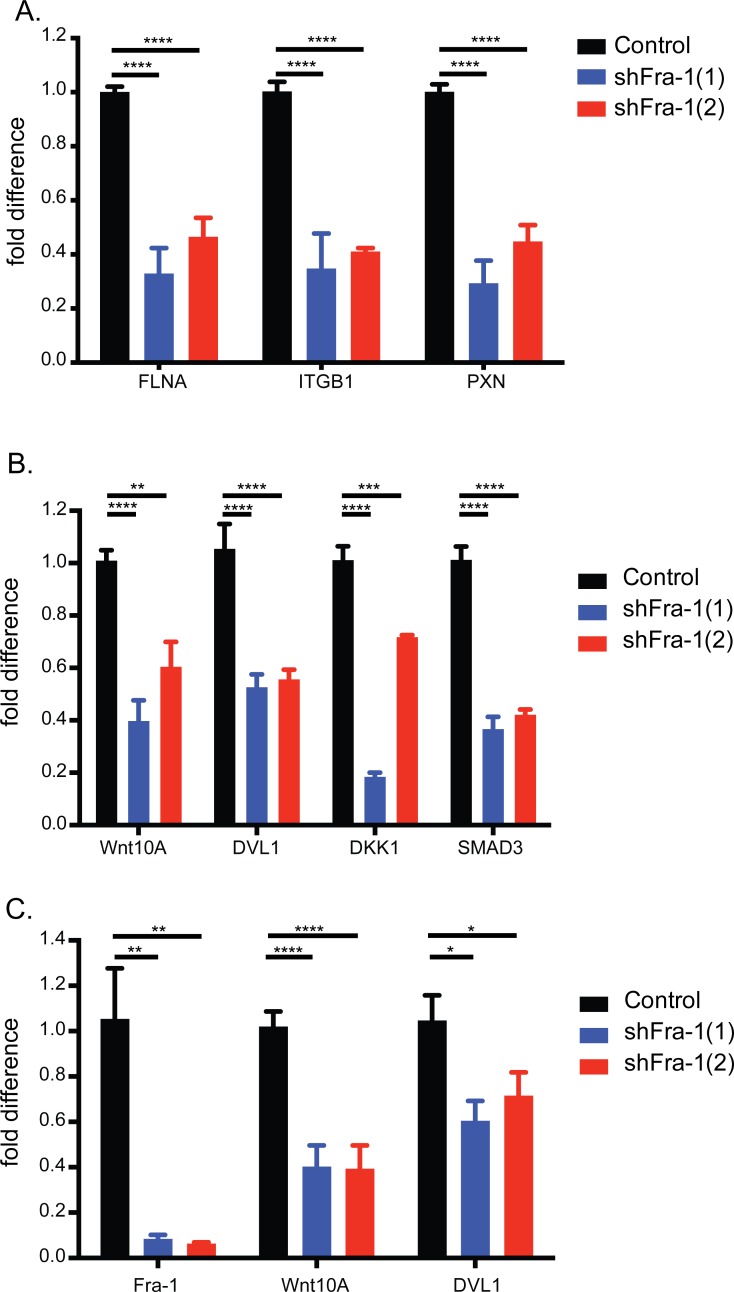
Fra-1 regulates the expression of Wnt pathway components **A**. Validation of the focal adhesion pathway by qRT-PCR upon Fra-1 depletion in HT29 cells. **B**.-**C**. qRT-PCR analysis of Wnt pathway components in the classifier in HT29 and HCT15 cells. Error bars represent SEM. Statistics: One_Way ANOVA * *p* < 0.05, ** *p* < 0.01, *** *p* < 0.001, ****p < 0.0001.

The influence of focal adhesions on motility and invasiveness and focal adhesion pathway regulation by Fra-1 in colon cancer cells are previously reported mechanisms of the pro-metastatic activity of Fra-1 [18], [25]. Consistently, Fra-1 knockdown in colon cancer cells decreased the expression of a panel of focal adhesion genes, indicating that our classifier is relevant and a reliable indicator of the aggressiveness of colon cancer (Figure 6A).

**Figure 7:**
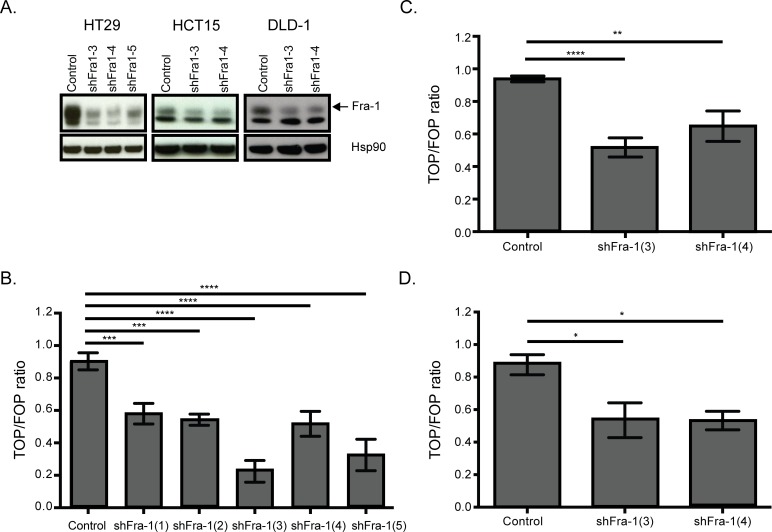
Fra-1 regulates beta-catenin activity **A**. Western blots showing efficient depletion of Fra-1 in HT29, HCT15, and DLD-1 cells. **B**.-**D**. Beta-catenin reporter assay on HT29, HCT15 and DLD-1 cells upon Fra-1 depletion. Error bars represent SEM. Statistics: One_Way ANOVA * *p* < 0.05, ** *p* < 0.01, *** *p* < 0.001, ****p < 0.0001.

On the other hand, regulation of the Wnt pathway by Fra-1 is an unexplored phenomenon. The Wnt pathway is significantly represented by seven genes in the classifier: whereas Wnt10A, SMAD3, DKK1 and DVL1 were downregulated upon Fra-1 knockdown, BAMBI, ROCK2 and PLCB4 were upregulated (Table 2). Notably, Wnt10A is the most abundantly expressed Wnt gene in HT29 cells (Suppl. Fig. 6). We validated Wnt10A, SMAD3, DKK1 and DVL1 dowregulation by Fra-1 depletion in HT29, and Wnt10A and DVL1 in HCT15 cells (Figure 6B-6C). We also examined by a luciferase reporter assay whether Fra-1 depletion modified beta-catenin activity. HT29, HCT15 and DLD-1 cells with or without a Fra-1 knockdown were transfected with the TOP/FOP constructs to measure transcriptional activity of beta-catenin upon loss of Fra-1 expression. We observed an effective reduction in the beta-catenin-mediated transcription between control and Fra-1-depleted cells (Figure 7). These data suggest that Fra-1 regulates the canonical Wnt signaling by modulating the expression of Wnt pathway components and the transcriptional activity of beta-catenin.

**Table 2:** KEGG pathway analysis on the classifier genes reveals Wnt pathway as one of the significantly regulated pathways by Fra-1

Term	p-value	Genes	Benjamini
Focal adhesion	0.0021	CAV2, CAV1, LAMB3, LAMA3, ROCK2, ITGA1, CAPN2, ITGB1, FLNA, PXN	0.1548
Axon guidance	0.0101	NRP1, ROCK2, ABLIM3, SEMA7A, ROBO2, EPHB3, ITGB1	0.1812
Hedgehog signaling pathway	0.0458	BMP4, WNT10A, GLI2, SHH	0.5231
Wnt signaling pathway	0.0648	WNT10A, PLCB4, DKK1, ROCK2, SMAD3, DVL1	0.5302
Leukocyte transendothelial migration	0.0886	ROCK2, NOX1, ESAM, ITGB1, PXN	0.5194

## DISCUSSION

The high lethality rate of colon cancer is mainly due to recurrence and distant metastasis. It therefore is crucial to better understand and predict these outcomes in order to take appropriate action with regard to treatment options. In this report, we demonstrate that Fra-1 is a critical biological determinant of colon cancer metastasis, as judged by two main observations. First, Fra-1 depletion severely impaired metastatic foci formation of colon cancer cells *in vivo*. Second, gene expression analysis by RNA sequencing of metastatic colon cancer cells revealed that a Fra-1 classifier comprising genes significantly regulated by Fra-1 is a strong predictor of disease-free survival.

Others have previously shown that Fra-1 is responsible for migration of colon cancer cells *in vitro* [18]. We found that Fra-1 is critical for the metastatic spread of colon cancer cells, even after establishment, yet largely dispensable for primary tumor growth. The growth rate of subcutaneous xenografts mirrors the results of 3D colony formation assays *in vitro*, showing a significant three-fold decrease in growth and colony number, respectively. These results, combined with the fact that Fra-1 knockdown was retained until the end of the experiment, indicate that Fra-1 is not critically required for primary tumor growth, since the tumors still grow in the absence of Fra-1. This differs, for example, from our recent observations for DDR kinases, for which shRNAs were commonly lost during tumor expansion [26]. In contrast, in an experimental metastasis model where the cells are injected intravenously, Fra-1-depleted colon cancer cells show a stark defect in their ability to form metastatic foci in the mice. The observation that the few tumor foci that could be found in the lungs of mice injected with Fra-1 depleted cells were largely Fra-1-positive indicate that the tumor burden is mainly caused by Fra-1-proficient cells. We did not observe such a negative selection pressure against, nor a similar growth disadvantage of, Fra-1-depleted cells in primary tumor growth. These data together suggest that Fra-1 has predominantly a metastasis-related role in colon cancer.

Metastasis is a stepwise process in which the cells must first disseminate from the primary tumor, join the blood stream or the lymphatic system by digesting through the stroma and the basement membrane, extravasate at a secondary site, and grow out [27], [1]. Since our experimental metastasis model bypasses the initial steps of metastasis and the cells directly enter the blood circulation upon injection, the difference between the metastatic ability of Fra-1-deficient and Fra-1-proficient cells is most likely because Fra-1 deficient cells fail at survival in the blood stream or at extravasation at a secondary site. By using an inducible system, and therefore giving equal chances of survival after inoculation, we tested whether the cells would still suffer from an acute loss of Fra-1 after intravenous injection and establishment of (micro)metastases. Extravasation and micrometastasis formation have been shown to occur within the first 24 hours after inoculation [28-30]. In the absence of the support of other cancer cells or a stromal mimic like matrigel, HT29 cells depleted of Fra-1 were 56 times less successful in forming metastatic tumor foci, resulting in significantly fewer and smaller tumor foci in the lungs of the mice. Heterogeneous Fra-1 levels in the tumor foci were commonly observed, with many cells showing restoration of Fra-1 levels, suggesting a negative pressure against Fra-1 knockdown cells. These results demonstrate further that Fra-1 is critical for the metastatic growth of colon cancer cells.

Fra-1 is overexpressed in several cancers [31] and we show that its expression correlates with a poor 5-year survival chance of colon cancer patients. It has proven difficult to develop inhibitors against transcription factors, making Fra-1 an unlikely drug target, even though this could aid in improving the treatment options of colon cancer patients. Furthermore, expression levels of transcription factors do not necessarily reflect the level of their activity. It has been suggested that in a data-driven approach, targets acting downstream of a transcription factor, rather than the transcription factor itself, possess better distinguishing features, because they reflect the activity of the transcription factor [32]. For these reasons, we compared the RNA expression profiles of Fra-1 proficient and deficient HT29 cells. We found a total of 199 genes significantly regulated by Fra-1. Our Fra-1 classifier is able to stratify colon cancer patients into three groups based on their disease outcome: two good (subtypes 2 and 3) and one poor (subtype 1) prognosis groups. Consistent with the role of Fra-1 in metastasis, in patients with a poor prognosis, genes positively regulated by Fra-1 are overexpressed while genes negatively regulated by Fra-1 have a low expression. Based on this classifier, disease-free survival rates of patients in subtype 1 are significantly lower than those in subtypes 2 and 3. The same pattern is observed for disease-specific and overall survival rates, however with less significance most likely owing to the fact that this information is only available for a subset of patient samples.

Fra-1 is a transcription factor functioning in heterodimers with other components of the AP-1 family. We and others have previously shown that Fra-1 down-regulation restores epithelial characteristics, including an epithelial-like morphology from a mesenchymal-like one, in breast cancer cells [7], [22] and colon cancer cells [33]. Moreover, several attempts to classify CRC based on gene expression data identified an EMT-related subtype associated with poor prognosis [34-37]. However, in colon cancer cell lines, we did not observe any change in the morphology, nor in E-cadherin or Vimentin protein levels upon Fra-1 depletion. We also failed to find any EMT genes significantly regulated in our RNA sequencing data. Furthermore, our colon cancer Fra-1 classifier has minimal or no overlap with other prognostic classifiers [35-39] nor with our Fra-1 breast cancer classifier [7]. Since Fra-1 has hundreds if not thousands of target genes, it is conceivable that it regulates several oncogenic processes via different mechanisms in different contexts. Furthermore, the basal expression pattern of Fra-1 target genes is conceivably not identical among different (cancer) tissues. On the other hand, earlier studies comparing metastatic to primary tumors to identify prognostic metastasis genes failed to identify Fra-1 [40-42], although Fra-1 was found to be upregulated in cancer cells compared to normal colon [40].

KEGG pathway analysis on this list of genes in the classifier revealed the focal adhesion and the Wnt pathways as overrepresented. Focal adhesions are known to be regulated by several AP-1 components, including Fra-1 [18], [43]. We confirmed the reliability of our results by validating the Fra-1-mediated regulation of several genes involved in focal adhesions by qRT-PCR. While expression of AP-1 components has been reported to be regulated by the Wnt pathway [10], [12], a reciprocal regulation between the AP-1 transcription factor complex and Wnt signaling has only been shown in an RNA profiling study [33] and awaits further validation. Here, we validated that Wnt pathway genes such as DKK-1, DVL-1 and Wnt10A are indeed positively regulated by Fra-1 in both HT29 and HCT15 colon cancer cell lines. Wnt10A plays an oncogenic role in renal cell carcinoma by activating the canonical Wnt pathway [44], and has been found to be highly expressed especially in the invasive fronts of esophageal cancer [45]. DVL-1 is a scaffolding protein that interacts with the Wnt receptor upon ligand binding and prevents the destruction of beta-catenin, allowing it to be transported to the nucleus and to form a transcription factor complex with TCF/LEF [46]. Despite some conflicting reports about DKK-1 promoting migration and invasion [47], it is recognized as a tumor suppressor and an inhibitor of Wnt signaling [48]. In this context, one would expect DKK-1 levels to increase upon depletion of Fra-1. However, since DKK-1 expression is regulated by beta-catenin [49], reduced beta-catenin activity results in reduced DKK-1 levels in Fra-1-depleted colon cancer cells. Although we did not see beta-catenin being directly regulated by Fra-1 at the RNA level, reporter assays showed that the activity of beta-catenin is decreased upon Fra-1 depletion. Because beta-catenin is known to regulate EMT and metastasis, it is plausible that the pro-metastatic function of Fra-1 is partially dependent on beta-catenin activity, which is tightly regulated by the Wnt pathway.

In conclusion, we find that Fra-1 is a critical factor in driving metastasis of human colon cancer cells *in vivo*. Furthermore, we show that a Fra-1 classifier is a highly significant predictor of patient outcome, independent of disease stage. We propose that Fra-1-regulated genes may be explored as therapeutic targets for colorectal cancer.

## MATERIALS AND METHODS

### Cell culture

HEK293T and colon cancer cell lines (HT29, HCT116, HCT15, DLD-1, Colo205) were cultured in DMEM supplemented with 2mM glutamine and 9% fetal bovine serum (Gibco). For lentivirus production, HEK293T cells were refreshed with complete medium containing 25 mM chloroquine, transfected with 8 μg of lentiviral construct and 4 μg of pMDLglpRRE, pHCMV-G, and pRSVrev and refreshed in complete medium after 6-8 hours. The lentivirus containing supernatant was used to transduce cell lines, followed by antibiotic selection when applicable. shRNA sequences were as follows: shFra-1(1): GTAGATCCTTAGAGGTCCT, shFra-1(2): GGCCTGTGCTTGAACCTGA, shFra-1(3): TRCN0000019539, shFra-1(4): TRCN0000019541, shFra1-(5): TRCN0000019542 (TRC Library, Sigma). The shRNAs 1 and 2 against Fra-1 were custom designed and cloned into KH vector. The others were cloned in the pLKO-puro vector. Fra-1 overexpression plasmid was from the CCSB-Broad lentiviral expression library in pLX204-Blast-V5 vector (Thermo Scientific). Luciferase expressing cells were generated by HIV-CS-CG-luc construct. shFra1-2 sequence was cloned into pLKO-teton vector to generate the inducible shFra1 construct. Cells harboring an empty pLKO-teton vector were used as control. pRL, TOP and FOP constructs for the beta-catenin reporter assay were a kind gift from Emile Voest, NKI. 2D proliferation assays were carried out by seeding 3*10^4^ cells/well in 6-well plates (Corning) and stained with crystal violet after 7 days. For 3D proliferation assays, 2.4*10^4^ cells were seeded in 0,4% low-melting-point agarose (Sigma) on 6-well plates (Corning) coated with a 1% agarose layer. The plates were stained with crystal violet after 3 weeks and colonies were counted by Image J software. Anoikis resistance experiments were performed on ultra-low-attachment 6-well plates (Corning). 0.4*10^6^ cells were seeded in complete medium. After 6 days, the cells were collected, washed, trypsinized, resuspended in complete medium and counted, or were collected and lysed for total protein measurement.

### Beta-catenin reporter assay

The cells were seeded on 24-well plates (Costar) at 1*10^5^ cells/well and co-transfected with 100ng of TOP or FOP constructs and 10ng of pRL using Lipofectamine 3000 reagent (Invitrogen) following manufacturers instructions. 48 hours after transfection, the cells were lysed and luciferase signals were measured in triplicates by dual-luciferase reporter assay (Promega). Transfection efficiencies were normalized by dividing the firefly luciferase signal by renilla luceriferase signal for each well. TOP/FOP ratio was calculated by dividing luciferase signal from TOP-transfected cells to FOP-transfected cells.

### Immunoblot analysis and antibodies

Cells were harvested by scraping in cold PBS and the pellets were lysed in RIPA buffer (50 mM TRIS pH 8.0, 150 mM NaCl, 1% Nonidet P40, 0.5% sodium deoxycholate, 0.1% SDS, complete protease inhibitor cocktail (Roche), and phosphatase inhibitors 10 mM NaF, 1 mM Na_3_VO_4_, 1 mM sodium pyrophosphate, 10 mM beta-glycerophosphate). After centrifugation the protein concentrations were determined by Bio-Rad protein assay (Bio-Rad). Immunoblot analysis was performed by using 4-12% Bis-Tris polyacrylamide-SDS gels (NuPAGE) and transferring these on to nitrocellulose membranes (Amersham). Membranes were blocked in 4% skimmed milk powder dissolved in 0,2% Tween-containing PBS and incubated with primary antibodies followed by secondary antibodies (Invitrogen). Primary antibodies used were Fra-1 (sc-605, Santa Cruz Biotechnology), human-specific Fra-1 (sc-28310, Santa Cruz Biotechnology), beta-actin (A5316, Sigma), Hsp90 (sc-7947, Santa Cruz Biotechnology).

### qRT-PCR primers

Total RNA was isolated by harvesting the cells in Trizol (Invitrogen) 6 days after lentivirus transduction and extracting RNA by subsequent chloroform, isopropanol and ethanol treatments. Following DNase treatment for 1 hour at 37°C, cDNA was prepared by a reverse transcriptase kit (Invitrogen). The average values obtained from two independent experiments are presented.

The primer sequences are as follows:

**Table:** 

Wnt10A	forward	GGAGACTCGCAACAAGATCC
	reverse	AAAGCGCTCTCTCGGAAAC
DKK-1	forward	CCTTGGATGGGTATTCCAGA
	reverse	CCGGAGACAAACAGAACCTT
DVL-1	forward	GAGCTTGAGTCCAGCAGCTT
	reverse	CGGATGAGTCTGGATGAGGT
SMAD3	forward	ACACCAAGTGCATCACCATC
	reverse	GCGGCAGTAGATGACATGAG
PXN	forward	GCACAATCCTTGACCCCTTA
	reverse	GAGCCGTACACAGGTGATGA
FLNA	forward	AGCCTCAACGTCACCTATGG
	reverse	ACTTGACCTTGGACGCATCT
ITGB2	forward	TTCTCCAGAAGGTGGTTTCG
	reverse	AGCAGCCGTGTAACATTCCT

### Survival analysis

From the Gene Expression Omnibus, we downloaded five publicly available data sets (GSE17536 [41], GSE17537 [41], GSE14333 [42], GSE33113 [50] and GSE37892) with gene expression data from primary CRC samples. Samples contained in both GSE14333 and GSE17536 were removed from GSE14333. Disease-free survival and staging information was available for a total of 578 tumor samples contained in these data sets. Disease-specific survival and overall survival were only available for datasets GSE17536 and GSE17537, totaling 232 tumors. Differences in survival times were analyzed using the Mantel-Cox log-rank test as implemented in the *survival* package. We performed survival analysis combining all stages stratified by stage and gender, and stage-specific survival analysis.

### RNA sequencing and generation of the Fra-1 classifier

Fra-1 was depleted from HT29 cells with two independent short hairpins. RNA was isolated by using Trizol and sequenced on a HiSeq 2000 System (Illumina). Data are available at NCBI Gene Expression Omnibus with the accession number GSE69415­­ (http://www.ncbi.nlm.nih.gov). Data were analyzed using the R statistical environment [49]. Illumina sequencing data was processed using DESeq version 1.12 [49]. We derived a Fra-1 knockdown (Fra-1KD) gene expression signature comparing RNA sequencing data from HT29 cell lines without and with shRNA knockdown of Fra-1. Knockdown was performed using two different hairpins in triplicate. In total, three samples of the wild type cell line and two knockdown samples (each with a different hairpin) were sequenced. Genes were selected as differentially expressed if they were differentially expressed between wild type and Fra-1KD cells but not between replicates. Nominal p-values were corrected for multiple testing using the Benjamini-Hochberg procedure and corrected p-values < 0.1 were regarded as significant. We applied the Fra-1KD signature to the independent data set consisting of 578 samples described above. More specifically, we used non-negative matrix factorization (NMF) as implemented in the NMF package for R (http://dx.doi.org/10.1186/1471-2105-11-367) to cluster the samples into three subtypes. We compared diseases free survival between these clusters as described above.

### Tumor xenografts and bioluminescence analysis

All animal work was done in accordance with a protocol approved by the Netherlands Cancer Institute Animal Experiment Ethics Committee. Female NOD/SCIDIL2gamma mice aged 5-8 weeks were used for all *in vivo* experiments. 0,5*10^6^ cells were injected into the lateral tail vein in 150ul PBS or subcutaneously into both flanks in a 100ul 1:1 mixture of growth factor reduced matrigel and complete medium. Subcutaneous tumors were manually measured twice weekly by a caliper. For bioluminescence imaging, the mice were intraperitoneally injected with 15mg/kg D-luciferin (Caliper Life Sciences) 15 minutes prior to imaging. The mice were anaesthetized and imaged with 60 seconds of exposure time (binning=8). Tumor burden in individual organs were quantified by injecting the mice with D-luciferin five minutes prior to sacrifice, harvesting the organs and imaging in a PBS-luciferin mixture. The data was analyzed by Living Image software. For shFra-1 induction, mice were treated with 2mg/ml doxycycline in drinking water containing 10mg/ml sucrose.

### Immunohistochemistry

Histological sections and hematoxylin-eosin staining were performed using standard procedures. Paraffin sections were deparaffinized, rehydrated, pretreated in 0.1 mM sodium citrate pH 6.0, washed and incubated with peroxide. The tissue was incubated with primary antibodies for Fra-1 (1:200, sc-28310, Santa Cruz Biotechnology) or GFP (1:2000, Abcam). Secondary antibody was PowerVision (DPVB-999HRP, ImmunoLogic). Peroxidase activity was detected with Liquid DAB (K3468, DAKO).

### Statistical analysis

Comparisons of two experimental groups were analyzed with two-tailed student's t-test. One-Way ANOVA corrected for multiple comparisons (Holm-Sidak) was used to compare more than two experimental groups (Prism; GraphPad Software). Error bars represent standard error of the mean (SEM).

Multivariate analysis was performed by fitting a Cox proportional hazards model to estimate hazard ratios for the subtypes by stratifying for gender and tumor stage.

## SUPPLEMENTARY MATERIAL FIGURES AND TABLES


