# Supplementary material for: Fra-1 is a key driver of colon cancer metastasis and a Fra-1 classifier predicts disease-free survival
Source: Oncotarget. 2015 Dec 3;6(41):43146–61. doi: 10.18632/oncotarget.6454 (PMC4791222; doi:10.18632/oncotarget.6454)
Supplement: Supplementary file 1 [file oncotarget-06-43146-s001.pdf]

Fra-1 is a key driver of colon cancer metastasis and a Fra-1 classifier predicts disease-free survival

Supplementary Material

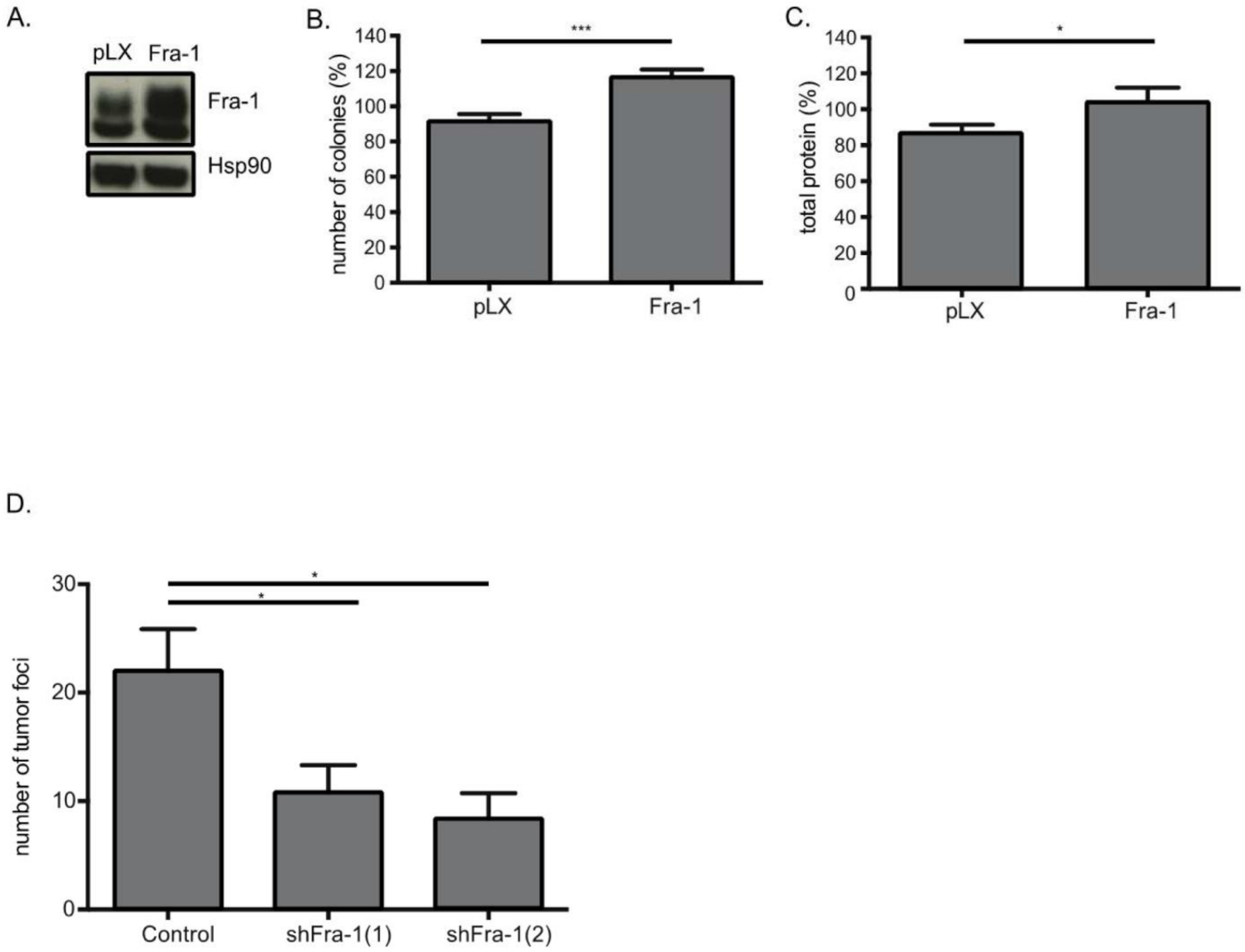

Supplementary Figure 1. A. Western blot of Colo205 cells overexpressing Fra-1. B. Colo205 cells with or without Fra-1 overexpression were seeded in triplicate into 0,3% agar suspension on top of a 1% agar base in 6-well plates at 24,000 cells/well. After three weeks, colonies were stained with crystal violet and counted by Image J software. C. $0,5*10^6$ HCT116 cells were injected intravenously into NSG mice ($n_{control}$=4, $n_{shFra-1(1)}$=5, $n_{shFra-1(2)}$=5). 5 weeks after injection, the mice were sacrificed and organs were harvested. Tumor foci in the livers were manually counted on H&E stained sections. Error bars represent SEM. Statistics: One-Way ANOVA * $p<0.05$

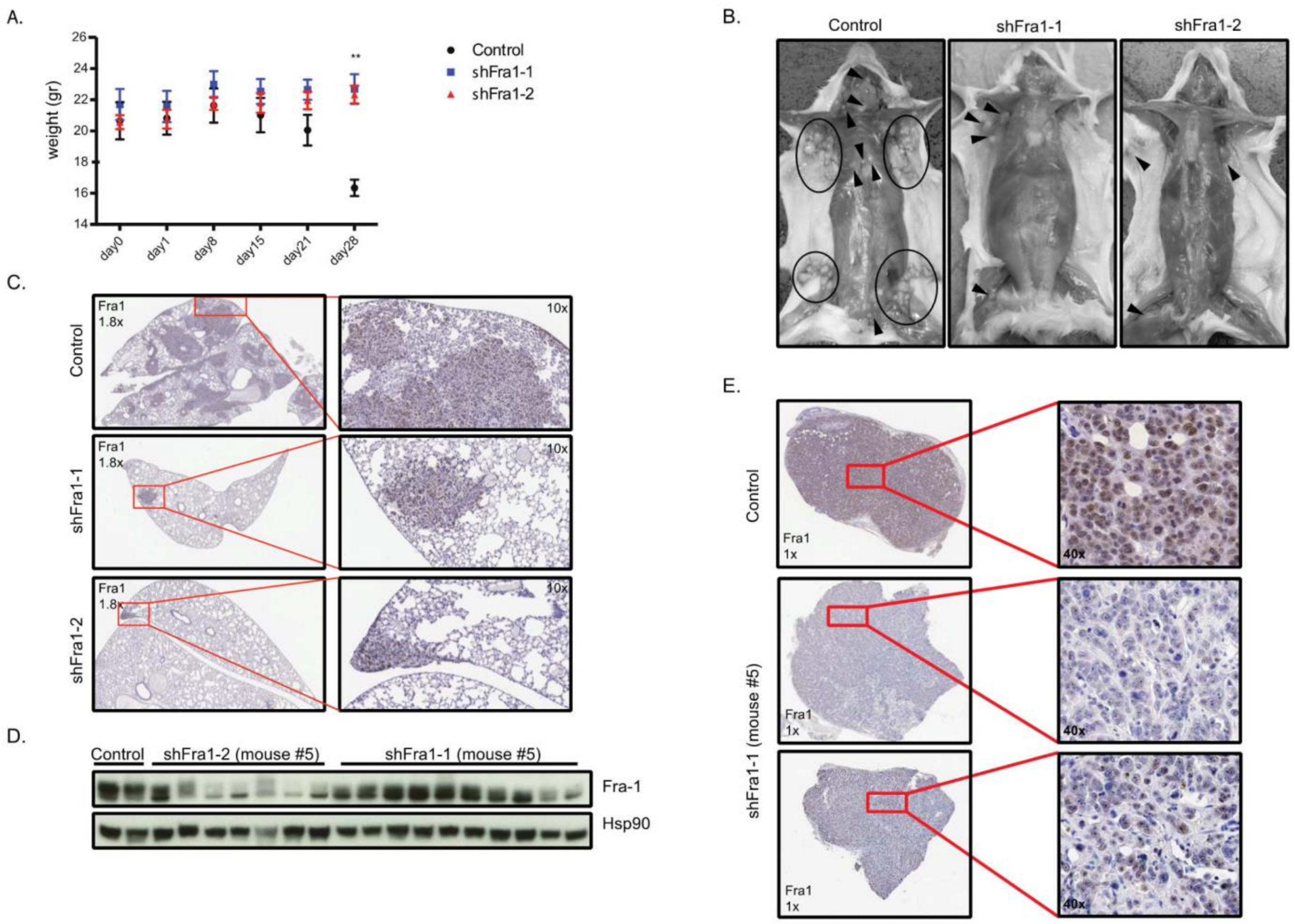

Supplementary Figure 2. A. Average weight of the mice in each group. B. Images of mice showing macroscoprically visible tumors on the subcutaneous skin and peritoneal wall. C. Fra-1 immunostaining on representative lung sections of mice injected with control or Fra-1-depleted cells. D. Western blots on tumors harvested from two representative mice injected with Fra-1-depleted cells. E. Fra-1 staining on tumor sections. Upper panel shows a Fra-1 positive control tumor. Lower panels show two tumors from the same mouse stained together on the same slide with heterogeneous Fra-1 staining.

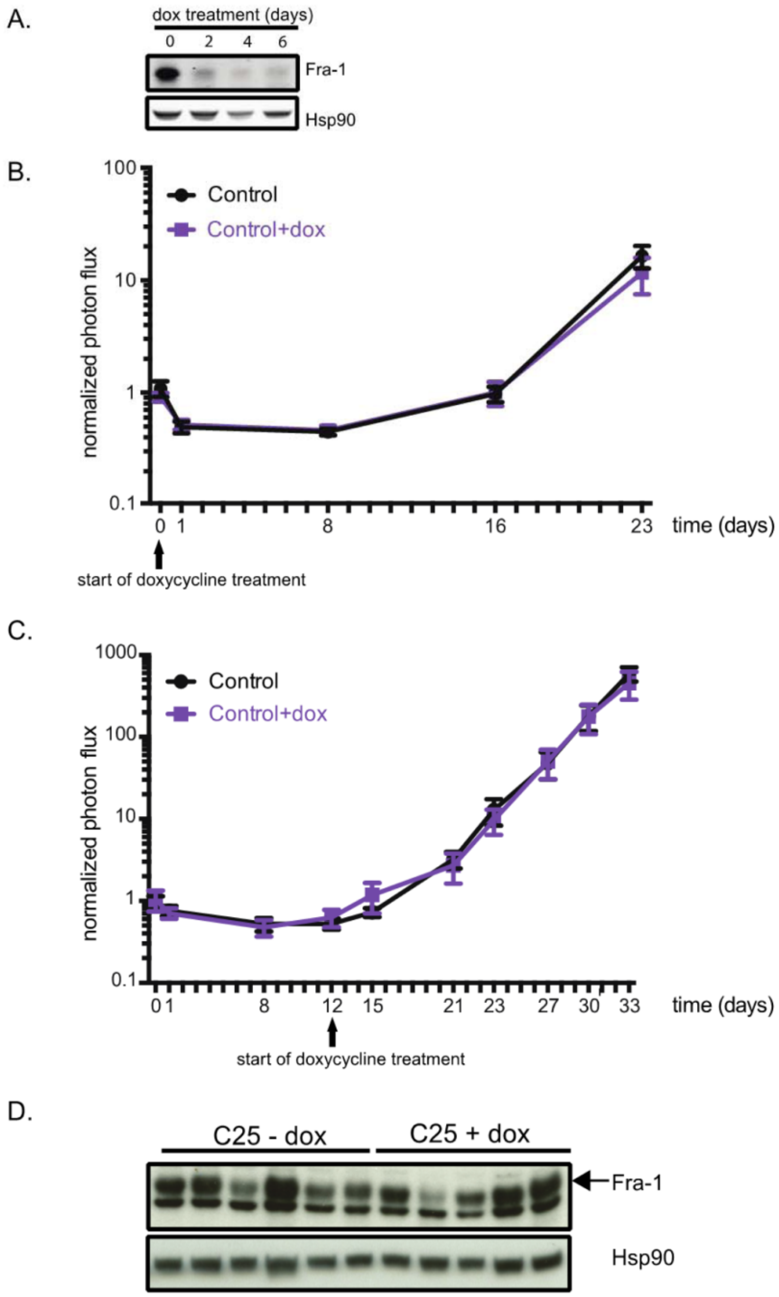

Supplementary Figure 3. A. Western blot showing C25 cells downregulating Fra-1 upon induction with doxycycline for 2, 4 and 6 days *in vitro*. B-C. Quantification of tumor expansion in mice injected with control cells and treated with doxycycline or not (n=6 per group). D. Western blots of tumors harvested from mice injected with C25 cells and treated with doxycycline or not.

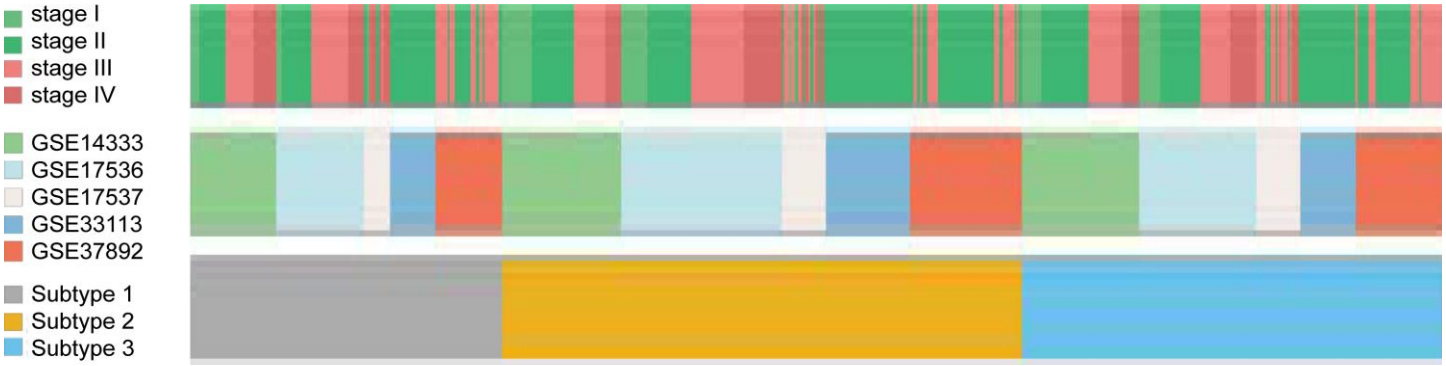

Supplementary Figure 4. The heterogeneity of the three subtypes with regard to tumor stage and dataset is shown in color coded bars.

A.

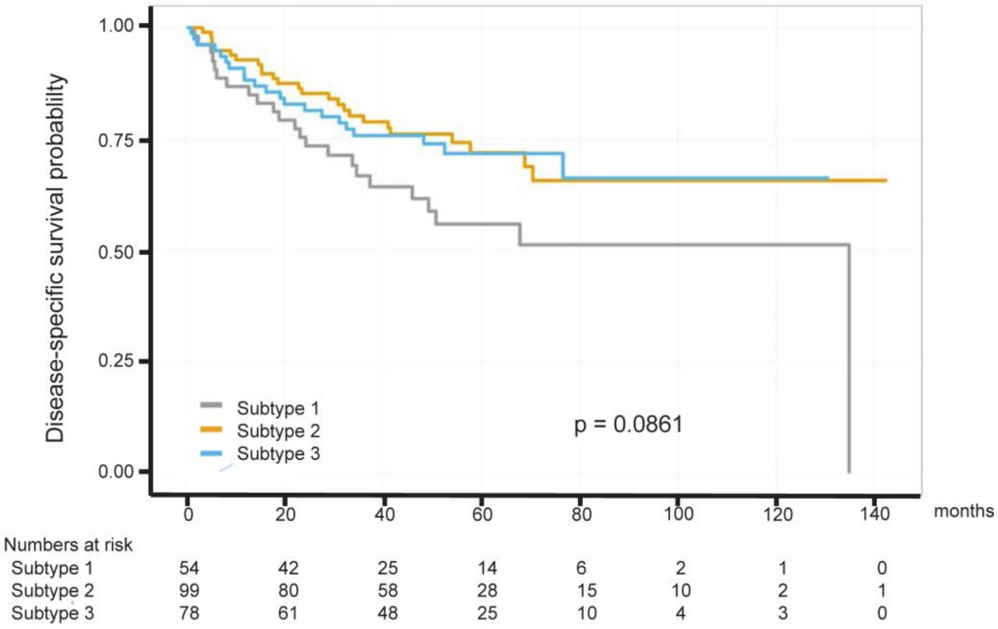

B.

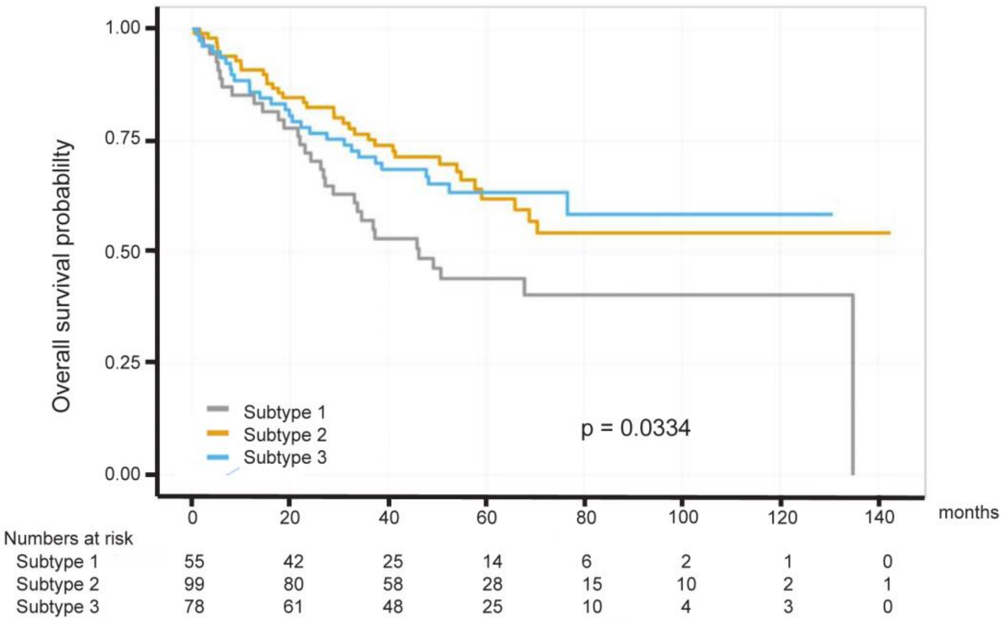

Supplementary Figure 5. A. Disease-specific survival curve for the three subtypes resulting from hierarchical clustering with the Fra-1 signature. **B.** Overall survival curve for the three subtypes resulting from hierarchical clustering with the Fra-1 signature.

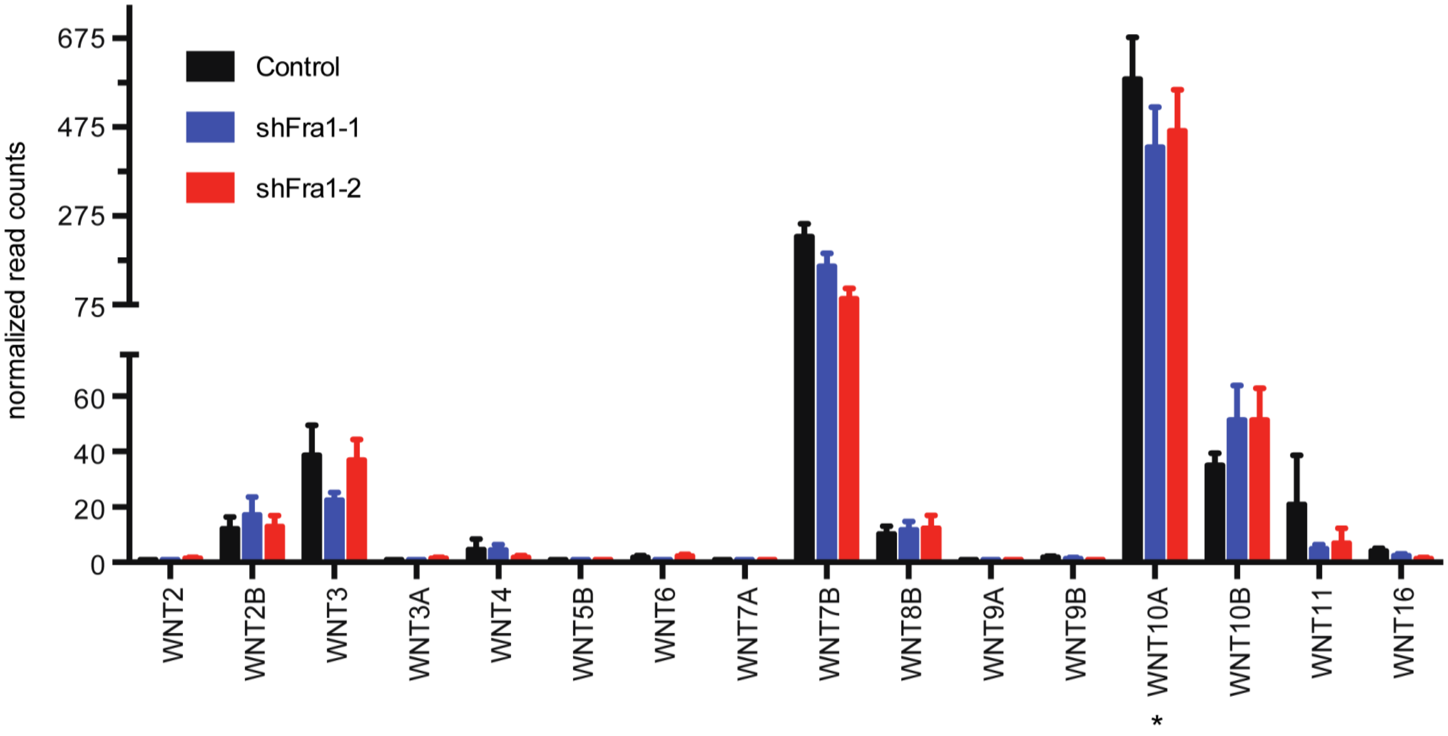

Supplementary Figure 6. Normalized read counts of all Wnt genes found to be expressed in HT29 cells with or without Fra-1 depletion.

Suppl. Table 1. List of genes in the classifier

Ensembl ID	Gene Symbol	Entrez ID	Fold Difference (log2)	FDR
ENSG00000144821	MYH15	22989	1.7556	9.40E-02
ENSG00000175592	FOSL1	8061	1.456	3.32E-06
ENSG00000123843	C4BPB	725	1.4271	1.41E-04
ENSG00000137440	FGFBP1	9982	1.3543	7.38E-04
ENSG00000107984	DKK1	22943	1.3463	1.89E-03
ENSG00000153292	GPR110	266977	1.3443	1.55E-05
ENSG00000214049	UCA1	652995	1.3021	1.32E-02
ENSG00000092929	UNC13D	201294	1.2329	4.58E-04
ENSG00000181126	HLA-V	NA	1.2052	7.99E-02
ENSG00000105974	CAV1	857	1.1673	8.53E-03
ENSG00000185567	AHNAK2	113146	1.146	7.84E-03
ENSG00000120708	TGFBI	7045	1.1016	4.50E-04
ENSG00000070404	FSTL3	10272	1.0556	2.35E-02
ENSG00000137878	GCOM1	145781	1.0251	5.31E-02
ENSG00000138623	SEMA7A	8482	0.9786	2.82E-02
ENSG00000075461	CACNG4	27092	0.9638	4.22E-04
ENSG00000111348	ARHGDIB	397	0.9593	5.79E-02
ENSG00000099250	NRP1	8829	0.9079	7.41E-03
ENSG00000196352	CD55	1604	0.9071	2.43E-09
ENSG00000167767	KRT80	144501	0.8855	5.97E-03
ENSG00000188042	ARL4C	10123	0.883	2.43E-02
ENSG00000167779	IGFBP6	3489	0.8526	3.15E-04
ENSG00000134954	ETS1	2113	0.8485	6.78E-04
ENSG00000177469	PTRF	284119	0.8171	1.75E-09
ENSG00000124225	PMEPA1	56937	0.8008	7.05E-04
ENSG00000147394	ZNF185	7739	0.783	3.05E-03
ENSG00000073756	PTGS2	5743	0.7782	3.15E-04
ENSG00000196154	S100A4	6275	0.7772	9.12E-02
ENSG00000117472	TSPAN1	10103	0.7478	2.72E-05
ENSG00000138119	MYOF	26509	0.7364	3.21E-04
ENSG00000154102	C16orf74	404550	0.7277	1.24E-02
ENSG00000114455	HHLA2	11148	0.7268	3.59E-02
ENSG00000150687	PRSS23	11098	0.674	1.41E-04
ENSG00000172927	MYEOV	26579	0.6616	3.94E-04
ENSG00000074047	GLI2	2736	0.6557	3.85E-02
ENSG00000171435	KSR2	283455	0.6521	2.89E-02
ENSG00000149564	ESAM	90952	0.6386	9.30E-02
ENSG00000164120	HPGD	3248	0.6218	1.70E-04
ENSG00000057019	DCBLD2	131566	0.6158	8.36E-03
ENSG00000103187	COTL1	23406	0.6093	7.71E-02
ENSG00000154217	PITPNC1	26207	0.5927	2.90E-04
ENSG00000088367	EPB41L1	2036	0.5848	1.80E-04
ENSG00000053747	LAMA3	3909	0.5819	7.84E-03

ENSG00000197757	HOXC6	3223	0.5722	5.55E-02
ENSG00000198825	INPP5F	22876	0.5694	4.95E-02
ENSG00000099810	MTAP	4507	0.5665	1.88E-03
ENSG00000197747	S100A10	6281	0.5648	3.05E-03
ENSG00000105971	CAV2	858	0.5647	5.37E-02
ENSG00000165388	ZNF488	118738	0.5596	1.45E-02
ENSG00000085788	DDHD2	23259	0.5557	5.98E-03
ENSG00000125378	BMP4	652	0.5449	7.41E-03
ENSG00000128567	PODXL	5420	0.537	3.38E-02
ENSG00000253368	TRNP1	388610	0.5345	8.02E-04
ENSG00000173210	ABLIM3	22885	0.5305	1.51E-02
ENSG00000205542	TMSB4X	7114	0.5246	1.60E-02
ENSG00000168785	TSPAN5	10098	0.5246	3.45E-02
ENSG00000142178	SIK1	150094	0.5221	9.93E-02
ENSG00000184916	JAG2	3714	0.5192	6.80E-02
ENSG00000225485	ARHGAP23	57636	0.5175	4.22E-04
ENSG00000107404	DVL1	1855	0.514	6.23E-03
ENSG00000188910	GJB3	2707	0.507	7.41E-03
ENSG00000127564	PKMYT1	9088	0.5041	2.96E-02
ENSG00000150782	IL18	3606	0.4957	2.37E-02
ENSG00000139211	AMIGO2	347902	0.4905	3.32E-03
ENSG00000118898	PPL	5493	0.4725	9.34E-03
ENSG00000100605	ITPK1	3705	0.4672	6.04E-03
ENSG00000106078	COBL	23242	0.4614	1.90E-02
ENSG00000162836	ACP6	51205	0.4499	1.87E-02
ENSG00000204619	PPP1R11	6992	0.4414	3.63E-02
ENSG00000196924	FLNA	2316	0.4402	7.84E-03
ENSG00000100504	PYGL	5836	0.4317	1.90E-02
ENSG00000135925	WNT10A	80326	0.426	5.55E-02
ENSG00000089159	PXN	5829	0.4138	1.90E-02
ENSG00000102007	PLP2	5355	0.4135	2.96E-02
ENSG00000141562	NARF	26502	0.4098	8.69E-02
ENSG00000166949	SMAD3	4088	0.404	3.18E-02
ENSG00000023171	GRAMD1B	57476	0.3945	3.54E-02
ENSG00000169894	MUC3A	4584	0.3929	7.21E-02
ENSG00000090621	PABPC4	8761	0.3922	2.75E-02
ENSG00000019505	SYT13	57586	0.3881	2.91E-02
ENSG00000198742	SMURF1	57154	0.3856	5.86E-02
ENSG00000196878	LAMB3	3914	0.3796	7.99E-02
ENSG00000150093	ITGB1	3688	0.3786	3.57E-02
ENSG00000131981	LGALS3	3958	0.3637	5.79E-02
ENSG00000152492	CCDC50	152137	0.3617	5.81E-02
ENSG00000162909	CAPN2	824	0.3579	6.70E-02
ENSG00000137309	HMGA1	3159	0.3572	4.95E-02
ENSG00000187109	NAP1L1	4673	0.3547	5.55E-02
ENSG00000187678	SPRY4	81848	-0.3772	8.42E-02
ENSG00000131389	SLC6A6	6533	-0.3927	5.11E-02

ENSG00000170145	SIK2	23235	-0.3932	4.65E-02
ENSG00000115993	TRAK2	66008	-0.3941	9.39E-02
ENSG00000106546	AHR	196	-0.4006	8.69E-02
ENSG00000134352	IL6ST	3572	-0.4087	3.45E-02
ENSG00000173702	MUC13	56667	-0.4091	7.21E-02
ENSG00000165156	ZHX1	11244	-0.4146	3.63E-02
ENSG00000134318	ROCK2	9475	-0.4164	3.45E-02
ENSG00000188559	RALGAPA2	57186	-0.4261	2.43E-02
ENSG00000145703	IQGAP2	10788	-0.4438	4.03E-02
ENSG00000085831	TTC39A	22996	-0.4583	7.48E-02
ENSG00000175311	ANKS4B	257629	-0.4642	9.30E-02
ENSG00000123636	BAZ2B	29994	-0.468	5.47E-02
ENSG00000021300	PLEKHB1	58473	-0.4681	2.43E-02
ENSG00000106351	AGFG2	3268	-0.4725	6.77E-02
ENSG00000111266	DUSP16	80824	-0.4808	5.37E-02
ENSG00000185127	C6orf120	387263	-0.4829	3.11E-02
ENSG00000184500	PROS1	5627	-0.4836	8.56E-03
ENSG00000116704	SLC35D1	23169	-0.4844	5.27E-02
ENSG00000126217	MCF2L	23263	-0.486	2.37E-02
ENSG00000177707	PVRL3	25945	-0.487	1.87E-02
ENSG00000161011	SQSTM1	8878	-0.4872	4.90E-02
ENSG00000170759	KIF5B	3799	-0.4895	1.88E-03
ENSG00000064270	ATP2C2	9914	-0.4905	7.47E-02
ENSG00000121064	SCPEP1	59342	-0.4927	8.34E-02
ENSG00000162174	ASRGL1	80150	-0.5113	1.86E-02
ENSG00000088992	TESC	54997	-0.5169	1.51E-02
ENSG00000151150	ANK3	288	-0.5193	3.71E-02
ENSG00000080546	SESN1	27244	-0.5259	3.71E-02
ENSG00000163590	PPM1L	151742	-0.5271	4.56E-02
ENSG00000145569	FAM105A	54491	-0.536	1.43E-02
ENSG00000077150	NFKB2	4791	-0.5559	1.77E-02
ENSG00000143153	ATP1B1	481	-0.561	1.05E-02
ENSG00000139318	DUSP6	1848	-0.562	6.52E-04
ENSG00000105854	PON2	5445	-0.5791	6.23E-03
ENSG00000148468	FAM171A1	221061	-0.5953	8.69E-02
ENSG00000164690	SHH	6469	-0.5961	7.21E-02
ENSG00000155629	PIK3AP1	118788	-0.6061	1.89E-03
ENSG00000169994	MYO7B	4648	-0.6141	2.79E-02
ENSG00000146802	TMEM168	64418	-0.6174	1.49E-04
ENSG00000164761	TNFRSF11B	4982	-0.6207	4.27E-02
ENSG00000234155		NA	-0.6213	3.85E-02
ENSG00000101333	PLCB4	5332	-0.6525	5.39E-02
ENSG00000170345	FOS	2353	-0.6596	6.52E-02
ENSG00000150961	SEC24D	9871	-0.6656	2.71E-03
ENSG00000138640	FAM13A	10144	-0.6767	1.48E-03
ENSG00000067113	PPAP2A	8611	-0.6779	1.32E-02
ENSG00000257743		NA	-0.6798	3.05E-03

ENSG00000168916	ZNF608	57507	-0.6803	4.65E-02
ENSG00000153071	DAB2	1601	-0.684	5.55E-02
ENSG00000069869	NEDD4	4734	-0.693	6.52E-02
ENSG00000172164	SNTB1	6641	-0.7117	1.43E-02
ENSG00000149212	SESN3	143686	-0.7138	5.85E-04
ENSG00000157388	CACNA1D	776	-0.7145	2.96E-02
ENSG00000072201	LNX1	84708	-0.7272	6.23E-02
ENSG00000081803	CADPS2	93664	-0.7289	3.45E-02
ENSG00000182489	XKRX	402415	-0.7472	1.24E-02
ENSG00000143416	SELENBP1	8991	-0.7594	1.28E-03
ENSG00000182580	EPHB3	2049	-0.7647	8.24E-04
ENSG00000123104	ITPR2	3709	-0.7733	3.57E-02
ENSG00000250722	SEPP1	6414	-0.7942	6.57E-02
ENSG00000110492	MDK	4192	-0.8022	6.74E-04
ENSG00000214290	C11orf93	120376	-0.8089	2.13E-02
ENSG00000165359	DDX26B	203522	-0.8128	4.90E-02
ENSG00000079385	CEACAM1	634	-0.8289	5.55E-02
ENSG00000168961	LGALS9	3965	-0.8363	2.85E-02
ENSG00000166750	SLFN5	162394	-0.8413	5.36E-03
ENSG00000118507	AKAP7	9465	-0.8434	4.38E-04
ENSG00000163072	NOSTRIN	115677	-0.8492	2.55E-04
ENSG00000135917	SLC19A3	80704	-0.8542	9.79E-02
ENSG00000114541	FRMD4B	23150	-0.8717	4.38E-04
ENSG00000167588	GPD1	2819	-0.8876	7.99E-02
ENSG00000225329		NA	-0.9024	6.78E-04
ENSG00000187134	AKR1C1	1645	-0.9215	4.72E-03
ENSG00000170608	FOXA3	3171	-0.9292	5.55E-02
ENSG00000007952	NOX1	27035	-0.9653	3.57E-02
ENSG00000049192	ADAMTS6	11174	-0.9711	2.35E-02
ENSG00000196139	AKR1C3	8644	-0.9803	4.15E-02
ENSG00000150893	FREM2	341640	-0.9818	3.81E-03
ENSG00000213949	ITGA1	3672	-1.0002	1.74E-06
ENSG00000137251	TINAG	27283	-1.0058	9.00E-04
ENSG00000095739	BAMBI	25805	-1.0344	3.32E-06
ENSG00000137699	TRIM29	23650	-1.0621	2.92E-02
ENSG00000107957	SH3PXD2A	9644	-1.0999	3.49E-02
ENSG00000152580	IGSF10	285313	-1.1255	5.86E-05
ENSG00000259974	LINC00261	140828	-1.228	1.83E-02
ENSG00000132329	RAMP1	10267	-1.2367	5.55E-02
ENSG00000106069	CHN2	1124	-1.2394	5.86E-05
ENSG00000122176	FMOD	2331	-1.2434	9.30E-02
ENSG00000197249	SERPINA1	5265	-1.2435	1.76E-02
ENSG00000031081	ARHGAP31	57514	-1.2731	1.85E-02
ENSG00000111885	MAN1A1	4121	-1.2758	1.66E-03
ENSG00000196167	C11orf92	NA	-1.3793	8.31E-02
ENSG00000132561	MATN2	4147	-1.4006	2.75E-02
ENSG00000132561	MATN2	100506558	-1.4006	2.75E-02

ENSG00000165092	ALDH1A1	216	-1.4059	3.63E-02
ENSG00000014257	ACPP	55	-1.4389	6.81E-04
ENSG00000222033		NA	-1.4424	5.56E-03
ENSG00000166922	SCG5	6447	-1.5011	9.30E-02
ENSG00000090920	FCGBP	8857	-1.5325	7.41E-03
ENSG00000185477	GPRIN3	285513	-1.5435	2.30E-02
ENSG00000151632	AKR1C2	1646	-1.6432	2.56E-03
ENSG00000151632	AKR1C2	101060798	-1.6432	2.56E-03
ENSG00000173467	AGR3	155465	-1.7722	2.14E-03
ENSG00000165029	ABCA1	19	-1.9713	8.24E-04
ENSG00000183091	NEB	4703	-2.0371	1.74E-06
ENSG00000160180	TFF3	7033	-2.0473	5.55E-02
ENSG00000122859	NEUROG3	50674	-2.0756	5.55E-02
ENSG00000172955	ADH6	130	-2.4258	8.53E-03
ENSG00000185008	ROBO2	6092	-4.1562	5.56E-02

Table S1. Genes comprising the Fra-1 classifier are listed with columns indicating their Ensembl ID, Gene symbol, Entrez ID, Fold difference (log2) and FDR.

Suppl. Table 2a. Cox Proportional Hazards Model for Disease-Specific Survival

		N(n)	HR	95% CI	p-value
**Subtype**	**1**	55 (24)	1		
	**2**	99 (25)	0.49	0.28 - 0.89	0.02
	**3**	78 (21)	0.74	0.39 - 1.39	0.35

Stage 2

		N(n)	HR	95% CI	p-value
**Subtype**	**1**	19 (5)	1		
	**2**	28 (0)	NA		
	**3**	25 (4)	0.68	0.17 - 2.72	0.58

Stage 3

		N(n)	HR	95% CI	p-value
**Subtype**	**1**	19 (8)	1		
	**2**	32 (5)	0.28	0.09 - 0.87	0.03
	**3**	25 (6)	0.66	0.23 - 1.93	0.45

Stage 4

		N(n)	HR	95% CI	p-value
**Subtype**	**1**	14 (11)	1		
	**2**	25 (19)	0.80	0.37 - 1.73	0.58
	**3**	17 (11)	0.68	0.289 - 1.61	0.38

Table S2a. Cox proportional hazards model for disease-specific survival estimating hazard ratios for the subtypes stratified for gender and tumor stage.

Suppl. Table 2b. Cox Proportional Hazards Model for Overall Survival

		N(n)	HR	95% CI	p-value
**Subtype**	**1**	55 (31)	1		
	**2**	99 (34)	0.52	0.32 - 0.86	0.01
	**3**	78 (28)	0.68	0.40 - 1.16	0.15

Stage 2

		N(n)	HR	95% CI	p-value
**Subtype**	**1**	19 (10)	1		
	**2**	28 (2)	0.15	0.03 - 0.68	0.01
	**3**	25 (4)	0.30	0.09 - 0.99	0.05

Stage 3

		N(n)	HR	95% CI	p-value
**Subtype**	**1**	19 (9)	1		
	**2**	32 (7)	0.36	0.14 - 0.98	0.05
	**3**	25 (12)	1.19	0.50 - 2.84	0.70

Stage 4

		N(n)	HR	95% CI	p-value
**Subtype**	**1**	14 (12)	1		
	**2**	25 (21)	0.76	0.37 - 1.55	0.455
	**3**	17 (12)	0.62	0.28 - 1.39	0.248

Table S2b. Cox proportional hazards model for overall survival estimating hazard ratios for the subtypes stratified for gender and tumor stage.
